# Establishing genetic manipulation for novel strains of human gut bacteria

**DOI:** 10.20517/mrr.2022.13

**Published:** 2023-01-03

**Authors:** Paul O. Sheridan, Ma’en Al Odat, Karen P. Scott

**Affiliations:** ^1^School of Biological and Chemical Sciences, University of Galway, Galway H91 TK33, Ireland.; ^2^Gut Health Group, Rowett Institute, University of Aberdeen, Foresterhill, Aberdeen, Scotland AB25 2ZD, UK.

**Keywords:** Gene transfer, conjugation, genetic manipulation, microbiota

## Abstract

Recent years have seen the development of high-accuracy and high-throughput genetic manipulation techniques, which have greatly improved our understanding of genetically tractable microbes. However, challenges remain in establishing genetic manipulation techniques in novel organisms, owing largely to exogenous DNA defence mechanisms, lack of selectable markers, lack of efficient methods to introduce exogenous DNA and an inability of genetic vectors to replicate in their new host. In this review, we describe some of the techniques that are available for genetic manipulation of novel microorganisms. While many reviews exist that focus on the final step in genetic manipulation, the editing of recipient DNA, we particularly focus on the first step in this process, the transfer of exogenous DNA into a strain of interest. Examples illustrating the use of these techniques are provided for a selection of human gut bacteria in which genetic tractability has been established, such as *Bifidobacterium*, *Bacteroides* and *Roseburia*. Ultimately, this review aims to provide an information source for researchers interested in developing genetic manipulation techniques for novel bacterial strains, particularly those of the human gut microbiota.

## INTRODUCTION

Genetic analysis is essential to our understanding of bacterial physiology and the role of specific bacteria in an ecosystem. Despite the dramatically increased interest in the human colonic microbial ecosystem and improved understanding of its important role in maintaining health, there has not been a similar increase in techniques to genetically manipulate these microorganisms. While genomic analysis can confirm the presence of a specific gene within a bacterium, understanding the gene’s function requires targeted manipulation. This is limited by our inability to introduce new DNA into most bacterial species. This knowledge gap is well recognised in the field of gut microbiota research and may soon generate a revival, which will combine classical genetic manipulation techniques with modern molecular biology and genomics. Genetic modification is also a first step in developing new biotechnological applications for a wide range of bacteria.

**Figure 1:**
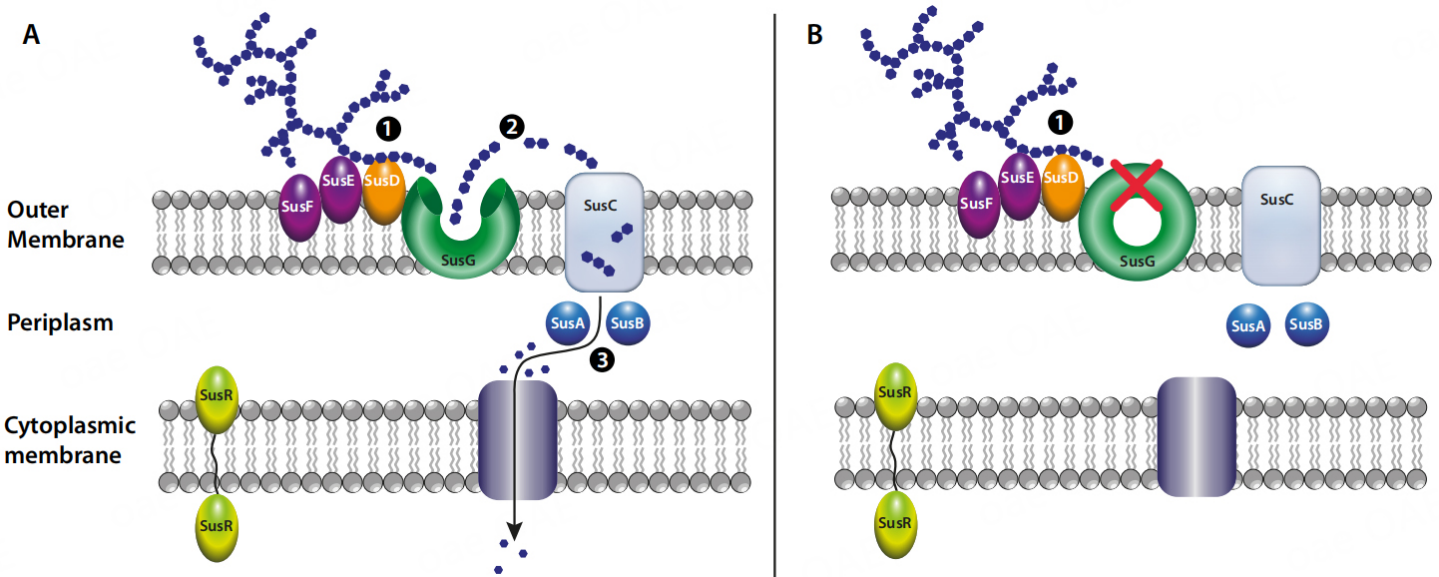
Molecular dissection of the Sus system of *B. thetaiotaomicron* involved in degrading dietary starch. When the SusG α-amylase enzyme is active (panel A), starch bound to the cell surface by starch-binding proteins is degraded, releasing oligosaccharides that are transported into the periplasm (SusC) and further degraded by periplasmic enzymes (SusA and SusB) prior to final uptake of monomeric units into the cell. When the susG gene is knocked out (panel B), starch bound to the cell surface is not degraded and there is no uptake and further degradation.

A striking example of how genetic manipulation techniques can revolutionize our understanding is presented in the human colonic *Bacteroides*. The gut microbiota of any adult is dominated by bacteria in the Bacteroidetes and Firmicutes phyla^[1]^. *Bacteroides* are one of the most dominant genera of anaerobic gut bacteria and have considerable metabolic diversity possessing many genes involved in polysaccharide degradation. Between 1976 and 1978, many researchers demonstrated the transfer of plasmids carrying antibiotic resistance genes between *Bacteroides* spp. and *E. coli*, using heat shock at 50 °C^[2,3]^ while in 1977, *Bacteroides thetaiotaomicron* was transformed using DNA from bacteriophage by calcium shock transformation^[4]^. The landmark research carried out in the development of these genetic manipulation techniques and their seminal use to fully characterise the mechanisms of starch utilization in *B. thetaiotaomicron*^[5]^ [Figure 1] resulted in several breakthroughs in our understanding of Gram-negative carbohydrate utilization^[6,7]^ and illustrated the opportunities of establishing these techniques for other species.

Introducing novel DNA into Gram-negative bacteria is facilitated by the cell wall structure consisting of an outer membrane and a thin peptidoglycan layer. In Gram-positive bacteria, although there is no outer membrane, the peptidoglycan layer is much thicker with more cross-linking and thus harder to weaken without destroying the bacterium. For this reason, there are far fewer publications describing successful genetic manipulation of Gram-positive bacteria, despite their importance and abundance in the human gut ecosystem^[1]^.

## GENETIC MANIPULATION OF GRAM-POSITIVE GUT BACTERIA

The importance of gut bacteria in maintaining health through the production of important metabolites and interaction with other members of the gut microbiota and with the host is now well-established. However, the mechanisms behind the majority of these interactions have not yet been explored, and only a small number of Gram-positive bacteria have successfully been genetically manipulated. A combination of genome sequence information and insertion mutagenesis will enable the roles of specific genes in host interactions to be confirmed, while genetic engineering provides many new opportunities for the development of such bacteria as therapeutic agents^[8,9]^.

The *Roseburia* genus is anaerobic Gram-positive bacteria belonging to the Lachnospiraceae family of Firmicutes, and is closely related to *Eubacterium rectale*^[10]^. *E. rectale* is alternatively known as *Agathobacter rectalis* gen. nov^[11]^. Although this reclassification has been controversial, it is clear that *Roseburia* species and *E. rectale* are phylogenetically and phenotypically closely related^[12-14]^. The *Roseburia*/*E.rectale* groups combined constitute 5%-15% of gut microbiota, and utilize polysaccharides to produce the short chain fatty acid butyrate^[1]^. Many studies have shown that these bacteria may provide health benefits to humans, such as (1) protection against colon cancer; (2) type II diabetes; (3) ulcerative colitis; and (4) in general, they may help restore the equilibrium of the gut ecosystem^[15]^.

Conjugation has been used successfully to introduce both conjugative transposons and shuttle plasmids into bacteria in the *Roseburia* genus. The first experiments involving genetic manipulation of closely related bacteria demonstrated transfer of the conjugative transposon Tn*B1230* carrying the *tet*(W) tetracycline resistance gene as a selectable marker between rumen and human *Butyrivibrio fibrisolvens* isolates^[16]^. The large 50 kb conjugative transposon integrated into preferential insertion sites in the recipient genome. Another novel conjugative transposon, Tn*K10*, carrying *tet*(O/32/O), was transferable to both *B. fibrisolvens* and to *Roseburia inulinivorans* isolates^[17,18]^. Separately, a shuttle plasmid expressing the glycoside hydrolase family 16 β-(1,3-1,4)-glucanase gene from *Streptococcus bovis* JB1 was constructed in *E. coli* and subsequently transferred into *Eubacterium rectale* and *Roseburia inulinivorans* strains, resulting in heterologous expression of the β-glucan degrading enzyme^[19,20]^. In this case, the use of the shuttle vector, containing origins of replication active in Gram-positive and Gram-negative bacteria was essential to construct these β-glucanase expressing recombinant gut bacteria.

Other Gram-positive human gut bacteria that have been successfully genetically manipulated include *Lactobacillus* and *Bifidobacterium* species^[21,22]^ [Table 1]. *Bifidobacterium* is one of the most abundant bacterial species in the gastrointestinal tract of infants and maintained at levels ranging from 0%-20% of the gut microbiota in adults^[23]^, while *Lactobacillus* spp. are generally less abundant. Both are widely used as probiotic bacteria due to their associated health benefits, but work to establish the specific mechanisms through which they interact with the host has been hindered by a lack of genetic manipulation tools. A number of methods, including homologous recombination and transposon mutagenesis, utilising electroporation and conjugation, have been reported for *Bifidobacterium* spp. (see review^[24]^). A replicase isolated from a *B. breve* megaplasmid was incorporated into a stable low-copy-number shuttle vector and used to introduce an amylopullulanase enzyme into *B. longum*, conferring the ability to utilise pullulan for growth^[25]^. 

**Table 1:** Commensal human gut microbes that have undergone successful genetic manipulation

**Organism**	**DNA transfer technique**	**References**
***Bifidobacterium*** ** spp.**	Conjugation, electroporation	Reviewed by^[21],[24]^
***Lactobacillus*** ** sp.**	Conjugation, electroporation	Reviewed by^[21]^
***Bacteriodes*** ** spp.**	Conjugation, transformation	^[2],[3]^
***Roseburia*** ** spp.**	Conjugation	^[19]^ https://www.sciencedirect.com/science/article/pii/S1075996419301052
***Eubacterium rectale***	Conjugation	^[19]^ https://www.sciencedirect.com/science/article/pii/S1075996419301052
***Blautia*** ** spp.**	Conjugation	^[28]^ https://www.nature.com/articles/srep13282
***Butyrivibrio fibrisolvens***	Conjugation	^[16]^
***Faecalibacterium*** ** spp.**	Conjugation	^[28]^ https://www.nature.com/articles/srep13282

The potential for genetically modifying probiotic bacteria to introduce new, or upregulate existing, metabolic activities has also driven research into delivering novel DNA into different *Lactobacillus* species (for details on reclassification of this genus see^[26]^), frequently utilising shuttle vectors capable of replicating in both *E. coli* and *Lactobacillus* species^[27]^. Shuttle vectors were also used in untargeted mating experiments between a donor *E. coli* and mixed faecal microbiota recipient culture, generating erythromycin-resistant transconjugants of several different Gram-positive gut bacteria containing the introduced shuttle plasmid [Table 1^[28]^]. 

## GENERAL METHODS OF DNA TRANSFER

The first step in developing a genetic manipulation technique for a novel species is identifying an appropriate method to transfer exogenous DNA into the recipient. Traditionally random mutants were created by chemical or ultraviolet mutagenesis without the need for DNA transfer. However, these methods are laborious, unpredictable, and introduction of point mutations may not necessarily fully inactive a gene. Although these techniques may still have some utility, the transfer of specific exogenous DNA into a recipient is usually preferable.

**Figure 2:**
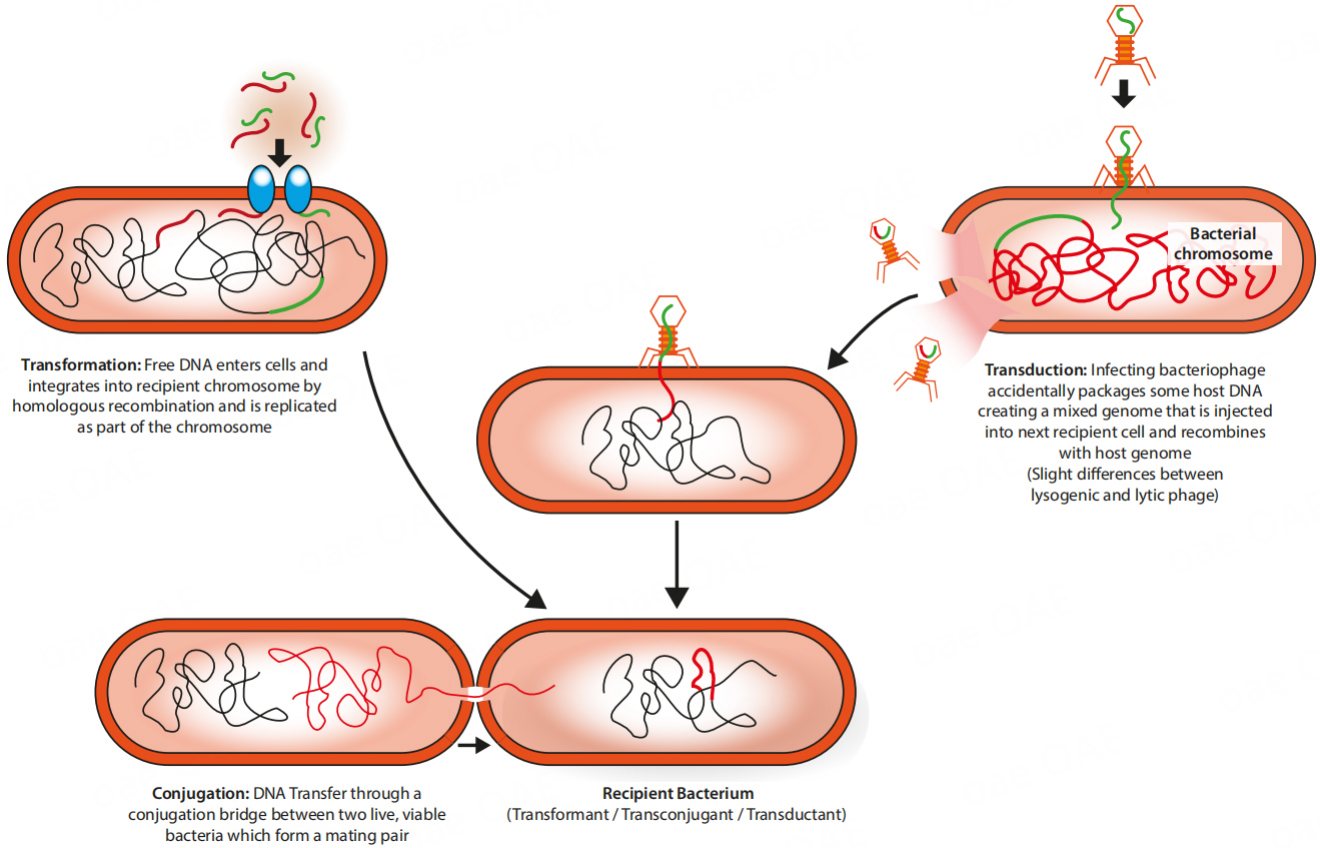
The three categories of exogenous DNA transfer into a bacterial cell.

There are three categories of exogenous DNA transfer into a recipient bacterium: (A) transformation, the direct uptake of DNA from an environment; (B) conjugation, the direct transfer of DNA from one cell to another; and (C) transduction, the introduction of DNA into a cell by a virus [Figure 2]. Several methods, with different pros and cons, exist for each of these categories [Table 2].

**Table 2:** Advantages and limitations of different methods of exogenous DNA transfer. Single-stranded DNA (ssDNA) and double-stranded DNA (dsDNA)

**Category**	**Method**	**Advantages**	**Limitations**
**Transformation**	Natural	Deliver as ssDNA and technically simple	Necessary genes only possessed by a small number of bacteria
	Chemical	High transfer efficiency and technically simple	Delivered as dsDNA and may lyse recipient cell membrane
	Electroporation	High transfer efficiency	Delivered as dsDNA and may lyse recipient cell membrane
	Biolistic	Novel	Delivered as dsDNA and requires expensive equipment
	Sonoporation	High transfer efficiency	Delivered as dsDNA
	Tribos	Novel and technically simple	Delivered as dsDNA
**Conjugation**	Conjugative transposons	Delivered as ssDNA and wide host range	Cell numbers cannot be recovered prior to chromosomal insertion, often have preferred insertion sites
	Suicide plasmid vectors	Delivered as ssDNA and easily customised in well characterised donor	Cell numbers cannot be recovered prior to chromosomal insertion
	Shuttle vectors	Delivered as ssDNA, easily customised in well characterised donor and transconjugant population recoverable	Can be difficult to eliminate from bacteria once established
**Transduction**		Very high transfer efficiency	Establishing for a new strain is very time consuming

### (A) Transformation

Transformation can be performed using six major methods: Natural, chemical, electroporation, sonoporation, biolistic and tribos transformation, with variable efficacies for different bacterial taxa.

**In natural transformation**, bacterial cells enter an internally regulated physiological state of “competence”, which allows them to uptake extracellular DNA. This process involves the activity of 20 to 50 proteins and is expressed in response to specific environmental conditions i.e., nutrient availability or cell density. The main advantage of natural transformation as a method of genetic manipulation is that the exogenous DNA is introduced to the cytoplasm as single-stranded (ssDNA)^[29]^, unlike other methods in which DNA enters in double-stranded form. The recipient restriction enzymes cannot act on ssDNA, but the recipient methylases, which protect DNA against restriction enzymes, do. This increases the chance that a methylase will have protected a given recognition site before the restriction enzyme can cut the reformed double-stranded DNA (dsDNA)^[30]^. Thus, transferring DNA into a cell in its single-stranded form increases its chance of survival. Another advantage of this method is that if the conditions that drive competence are known, it is relatively straightforward. However, only about 1% of the bacteria studied to date are known to be naturally transformable^[31]^, and the conditions that drive competence are often not known and vary greatly between species and strains^[32]^.

**Chemical transformation** involves inducing transformation by chemical means, most commonly using CaCl_2_ or polyethylene glycol (PEG) treatment to disrupt or remove the bacterial cell wall. This method has a high DNA transfer efficiency in some bacteria^[33,34]^. However, CaCl_2_ treatment works poorly outside of *E. coli* strains, and PEG transformation requires the formation of protoplasts, followed by regeneration of the cell wall, and many species may not survive in the process.

**Electroporation** is based on the observation that when bacterial cells are subjected to electric shock, they can uptake extracellular DNA^[35]^. This can be a highly efficient means of introducing exogenous DNA into a recipient cell but may lyse the cell membrane, thus killing the recipient. Sonoporation involves subjecting bacteria to ultrasonic treatment in the presence of extracellular DNA. It is believed to have several advantages over electroporation, including the fact that it does not require an ion-free medium and can therefore be applied directly to cells growing in optimal medium or in human fluids^[36]^. Both of these methods are theorised to disrupt the cell membranes by creating pores, enabling the passage of extracellular DNA, whose negative charge would usually prevent it from passing through the hydrophobic bilayer of the cell membrane.

**Biolistic and tribos transformation** are less established methods of transformation in bacteriology. Biolistic transformation is more common in plant genetics, but several experiments have used this method in bacterial species. This involves the precipitation of a plasmid onto tungsten or polyol gold particles^[37,38]^ that are then accelerated at high velocity towards recipient cells. This technique may provide a means of transforming a wide range of bacteria, as innovations in inorganic chemistry produce smaller and smaller plasmid-associated microcarriers, although the biolistic devices or particle inflow guns used to accelerate the microcarriers can be prohibitively expensive. 

Tribos transformation involves the generation of sliding friction between a colloidal solution containing nano-sized acicular (needle-like crystal) material, bacterial cells and plasmid DNA molecules by vigorous plating on agar. It is hypothesised that the plasmid DNA associates with the acicular material and this complex then penetrates the bacterial cell, introducing the plasmid DNA to the bacterial cytoplasm^[39]^. This method has been used successfully for *E coli* and *Bacillus subtilis*^[40]^*.*

### (B) Conjugation

Conjugation is the transfer of DNA from one cell to another cell by direct interaction between live bacterial cells. Conjugation bridges join a mating-pair of cells and transmembrane pores form, allowing DNA to be passed between the cells. As these interactions are temporary, usually only small genetic elements, such as plasmids or conjugative transposons (CTns), have time to transfer their entire DNA sequence into the recipient cells. If these genetic elements are capable of replication in the recipient cell or insertion into the recipient chromosome, they can be stably passed on to subsequent generations. There are three main vehicles to genetically manipulate a recipient bacterium via conjugation: CTns, suicide plasmid vectors and shuttle vectors.

**Conjugative transposons** combine features from transposons, plasmids and bacteriophages. They normally reside in a bacterial chromosome and are passively replicated during chromosomal replication. However, they can excise from the chromosome and form plasmid-like covalently closed circular transfer intermediates^[41]^. These intermediates are generally thought to be non-replicating, but exceptions have been found^[42-44]^. The chromosomal excision and integration of CTns resembles that of temperate bacteriophages, which also form circular intermediates. However, CTns do not form viral particles and are transferred via conjugation bridges, not by bacteriophage transduction^[41]^. CTns have been shown to transfer and insert into a wide range of hosts^[45,46]^, but tend to integrate into specific regions of the recipient chromosome, termed “hotspots”. This makes them useful to introduce new genes into recipient bacteria, i.e., “knockin” mutagenesis, but generally not to “knockout” functions of targeted genes. However, a derivative of the CTn Tn*916*, carrying a copy of the sigK gene from *C. difficile*, was conjugated from *B. subtilis* JH-B3 into *C. difficile* CD196 and, rather than inserting into the recipient chromosome at the normal hotspot of the wild-type Tn*916* transposon, it inserted within the endogenous *sig*K gene through homologous recombination^[47]^. Although designed as a knockin experiment, this demonstrated that conjugative transposons could be directed to different integration sites, making them possible tools for targeted knockout mutagenesis. 

One of the difficulties in establishing genetic manipulation techniques for novel bacterial species is often a lack of selectable markers, such as antibiotic resistance genes, that work well in recipient species. Promiscuous CTns harbour a wide variety of antibiotic resistance genes, which provide a useful resource of genes that confer a strong selectable antibiotic resistance phenotype in a broad range of organisms.

**Figure 3:**
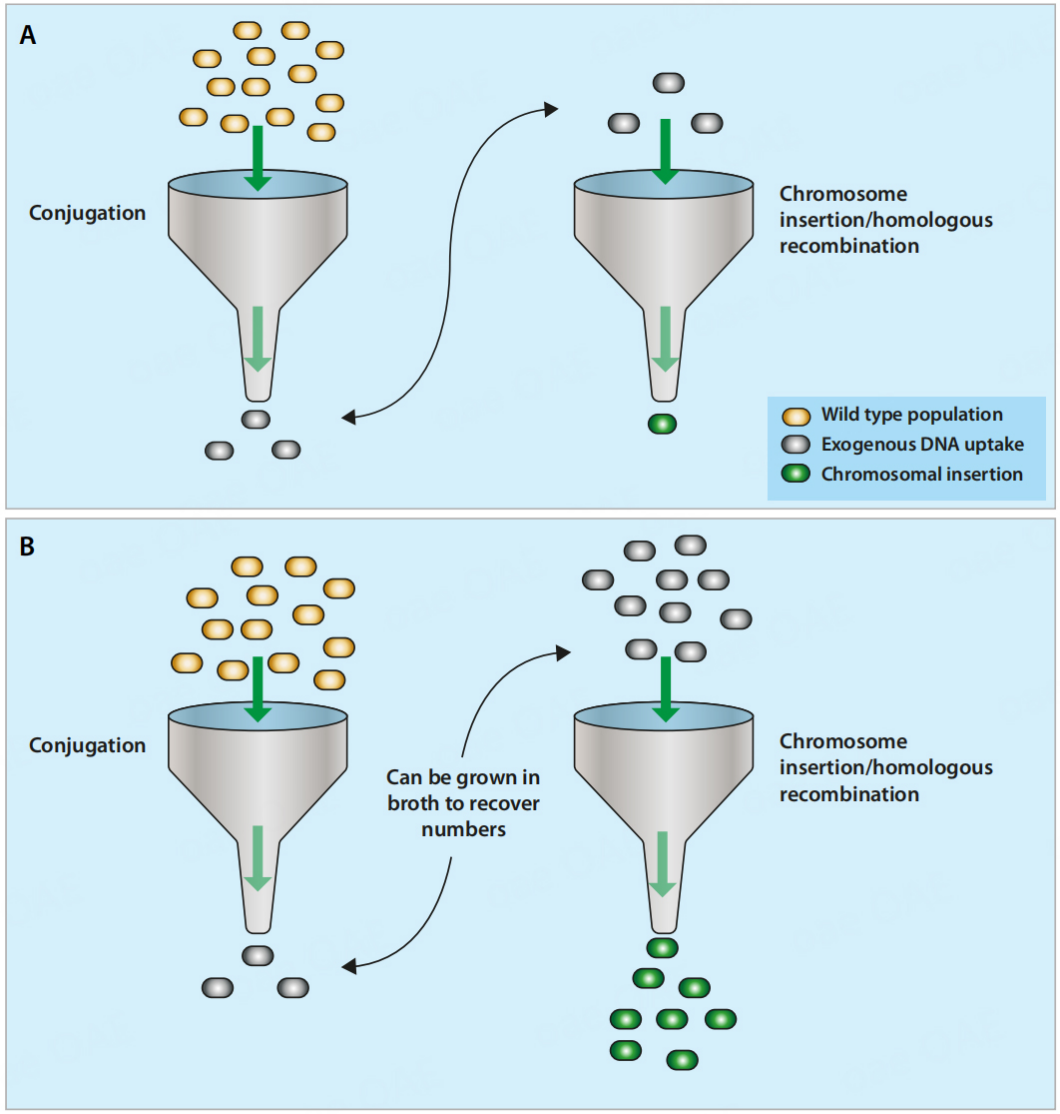
Bottlenecking effect. Only a small number of the initial recipient population become transconjugants, and only a small number of this population undergo chromosomal insertion or homologous recombination. (A) With CTns and suicide plasmids, the reduction in population is compounded during serial steps. (B) With autonomously replicating plasmids, the population can be recovered between conjugation and chromosomal insertion/homologous recombination. Initial wild-type population (yellow), transconjugants (grey) and chromosomal insertion generating desired mutants (green).

**Suicide plasmid vectors** are plasmids that are unable to replicate in the recipient strain but harbour a selectable marker that functions in the recipient. Thus, subjecting the recipient bacterium to selection (perhaps a specific antibiotic) limits growth to those bacteria that have integrated the plasmid into their chromosome, where the required gene can be expressed. In addition to a recipient-specific selection marker, conjugative suicide plasmid vectors require a selectable marker that functions in the donor. This could be the same gene, as many selectable markers work in a variety of bacterial species and strains. However, genes that confer an alternative resistance (e.g., to a different antibiotic) may intentionally be chosen, with one of the genes only functioning in the donor and the other only functioning in the recipient. This enables the donor to be counterselected after the mating step, facilitating the detection of transconjugants. Conjugative suicide plasmid vectors also require an origin of replication and an origin of transfer (*ori*T) that function in the chosen donor. If the donor strain is a bacterium with well-established molecular biology techniques (such as *E. coli*), the plasmids can be extensively customised, enabling the development of intricate genetic manipulation techniques, such as the generation of in-frame gene deletions. However, mutagenesis based on both CTns and suicide plasmid vectors suffers from a compounded reduction in the required transconjugant bacterial population [Figure 3], as only a small proportion of recipient bacteria take up the CTn/suicide plasmid vector, and of this small proportion, in only a small fraction will the incoming DNA undergo homologous recombination with the recipient chromosome. 

**Shuttle plasmid vectors** are similar in structure to suicide plasmid vectors. However, these plasmids either have an origin of replication that functions in both bacteria, or have two distinct origins of replication that allows the plasmid to replicate in both the donor and the recipient [Table 3]. In common with suicide plasmid vectors, shuttle vectors can be extensively customised in well characterised donors, facilitating intricate genetic manipulation techniques. The essential difference between this method and the other methods of conjugative transfer is the ability to recover the bacterial population of transconjugants after conjugation [Figure 3B]. This eliminates the bottlenecking effect, which hinders the efficiency of CTns and suicide plasmid vectors. It can however be difficult to eliminate replicating plasmids from a bacterium once they are no longer desired, after integration of the required fragment into the chromosome. This problem can be addressed by using intermediate-stability plasmids that are eliminated from the transconjugant population over several generations when selection for them is removed. Plasmid elimination can also be driven by plasmid-encoded negative selection markers, such as the *B. subtilis sacB* gene, whose activity is lethal to many Gram-negative bacteria growing in media containing high levels of sucrose^[48-51]^, or the *upp* gene, whose activity is lethal to Gram-positive bacteria growing in media containing 5-fluorouracil^[52]^. Plasmids that can only be maintained within a limited temperature range can also be selectively eliminated by heat curing. This method has been used to create insertion mutants in various *Bifidobacterium* species and requires lower transformation efficiencies. Following transformation with the plasmid containing the target gene fragment, increasing the growth temperature so that the plasmid cannot replicate creates active selection promoting recombination and the formation of insertion mutants^[53]^. 

**Table 3:** Plasmids with origins of replication known to function in alternative gut bacteria

**Genus of interest**	**Plasmid replicon**	**Source of plasmid**	**Additional replicative hosts**	**References**
***Prevotella***	pRR12	*Prevotella ruminicola* 223/M2/7	*P. ruminicola* 2202	[122]
			*Bacteriodes vulgatus* 1447	
			*Bacteroides uniformis* 1100	
	pB8-51	*Bacteroides eggerthii*	*Prevotella bryantii* B_1_4	[123]
	pRR17	*P. ruminicola* 23	*P. ruminicola* NCFB 2202	[124]
			*Bacteroides distasonis*	
			*Bacteroides thetaiotaomicron*	
			*B. uniformis*	
***Ruminococcus***	pRf186	*Ruminococcus flavefaciens* 186		[125]
	pAR67	*Ruminococcus albus* AR67		[126]
	pBAW301	*Ruminococcus flavefaciens *R13e2		[127]
	pAMβ1	*Enterococcus faecalis* DS5	*R. albus*	[128]
	pPSC22	*Lactobacillus plantarum*	*R. albus*	[129]
			Several *Lactobacillus* species	[130]
	pCK17	*Streptococcus lactis*	*R. albus*	[129]
***Roseburia***	pCB102	*Clostridium butyricum*	*Roseburia inulinivorans*	[19],[131]
			*Eubacterium rectale*	[19]
			Several *Clostridium* species	[132]
	pBP1	*Clostridium botulinum*	*Roseburia inulinivorans*	[19]
			Several *Clostridium* species	[132]

### (C) Transduction

Transduction involves the transfer of DNA from a bacteriophage into a recipient bacterium. Tailed bacteriophages are perhaps the most efficient means of exogenous DNA transfer that exist in nature, compacting 19-500 kb of dsDNA into the bacteriophage head^[54]^, and phages have evolved various means of avoiding expulsion by the infected bacterial cells^[55]^. Decades of detailed research behind bacteriophages have made them an attractive resource for the development of genetic manipulation techniques. Indeed, in species where transduction systems have been established, such as in *E. coli* and *Salmonella*, they have been shown to be highly efficient in transferring exogenous DNA into bacterial cells. However, the initial stage of infection (absorption) requires the interaction of a specific bacteriophage factor with a specific host cell component, termed the bacteriophage receptor. Because bacteriophage receptors vary dramatically between species and strains, the host range of a bacteriophage is extremely limited, and thus the same transducing bacteriophages are unlikely to work for a wide range of species and strains. It is also difficult and time-consuming to identify an appropriate transducing bacteriophage for a given bacterium. However, there could be instances, such as site-directed mutagenesis and killing of targeted bacterial pathogens, where the ability to specifically inactivate precise genes in a targeted bacterium would be an advantage.

Bacteriophages have also been shown to play a role in the transfer of antimicrobial resistance genes. Phage øC2, a bacteriophage infecting *Clostridiodes difficile* was able to transfer the mobilizable transposon Tn*6215* that carries an erythromycin resistance gene between *C. difficile* strains CD80 and CD062 at higher frequencies than observed by conjugative transfer^[56]^. Notably, the Tn*6215* transposon integrated at the same site in the recipient genome, independent of transfer method. 

## TECHNIQUES TO IMPROVE THESE METHODS

The general gene transfer methods described thus far have been used in a variety of bacterial species [Table 1], and those that result in the highest transfer efficiency have been shown to be highly species-specific. Such specificity arises from differences in the thickness and complexity of the peptidoglycan layer, different arrays of restriction-modification systems, differences in levels of natural competence and the ability of introduced DNA to be expressed in the new host. Thus, the most appropriate procedures for a given bacterium of interest must be determined by trial and error. The different procedures for electroporation and conjugation have been thoroughly investigated for selected bacterial species, resulting in specific refinements to the techniques to improve the transfer efficiencies [Table 4].

**Table 4:** Challenges in introducing exogenous DNA in novel microbes and methods to overcome them

**Challenge**	**DNA transfer technique**	**Solutions**
**Restriction of exogenous DNA by host R/M system**	Transformation	Methylation of DNA by commercially available methylases
		Methylation of DNA by cell-free extracts of the recipient strain
	Conjugation	Conjugation is inherently more resistant to exogenous DNA restriction
	General	Heat treatment of recipient
		DNA modification by passage through an intermediate recipient
		Plasmid artificial modification
**Low transfer frequencies**	Transformation	Larger amounts of exogenous DNA
	Conjugation	Smaller sized exogenous DNA fragments
		Facilitating close contact between donor and recipient
		Increasing donor:recipient ratio
	General	Autonomously replicating vectors (population recovery)
**Unsuccessful transfer**	General	Different counter-selection (e.g., antibiotic resistance gene)
		Different origin of replication in autonomously replicating vectors

### Improving transfer efficiency: electroporation

Electroporation consists of three stages: the preparation of electrocompetent cells, electropulsing and cell recovery. There have been numerous studies investigating and optimising each of these stages in a multitude of bacterial species to increase transfer efficiency, including a notably thorough investigation of all three stages of electroporation in *Staphylococcus carnosus*^[57]^.

In the preparation of electrocompetent cells, the bacterium of interest is harvested from a broth culture, usually in exponential phase. This culture is sometimes grown in glycine-rich medium to weaken the cell wall by replacing D-alanine which decreases peptidoglycan cross-linkages during cell wall synthesis^[58]^. This has been shown to increase transfer efficiency in members of the *Enterococcus*, *Lactococcus*, *Lactobacillus* and *Streptococcus* genera^[59-62]^. However, this increase in efficiency was not universal among Gram-positive bacteria, and the transfer efficiency actually decreased in *S. carnosus* when a glycine-rich medium was used^[57]^. 

During pulsing, electrocompetent cells are suspended in an electroporation buffer. Various additives (e.g., maltose, sucrose, mannitol, PEG 1500) and protocol tweaks have been shown to increase the transfer efficiencies of specific species, but these effects are highly species-dependent. The low transformation efficiencies of *Bifidobacterium* spp.^[63]^ were improved for *B. animalis* by adding 0.5 M sucrose to the MRS growth medium and washing buffer, and adding citrate to the electroporation buffer^[64]^. Transformation of *B. bifidum* was only possible after addition of 16% FOS or 10% GOS to the growth media and growing the bacterial cells to late log phase^[65]^. Electrotransformation efficiencies for *E. coli* TG1 were improved by increasing the culture time (8-10 hrs), the bacterial cell concentration (OD_600_ = 0.45) and the culture volume^[66]^. A novel way to increase the introduced culture volumes is to replace electroporation cuvettes with a simple M-TUBE device based on syringes and plastic tubing^[67]^. The use of the M-TUBE increased electroporation efficiencies for *E. coli* and *B. longum*, and this increased efficiency offers great potential in creating mutant libraries.

It should also be noted that although pulsing on ice, with ice-cold competent cells, is used routinely for most bacteria, pulsing at room temperature increased transfer efficiency in *Staphylococcus* species^[57,68,69]^, and various Gram-negative bacterial genera^[70]^. In contrast, extending the time cells were stored on ice prior to electroporation improved the transformation efficiency for bifidobacteria^[63]^. Following pulsing, cells are usually recovered by incubation in a recovery broth, a nutrient-rich medium, containing the components necessary for stabilising the stressed cell envelope, before selection of transformants. As not all bacteria have the same growth requirements, it can be difficult to determine the most appropriate recovery broth from a given bacterial species. However, early studies investigating the optimal transformation conditions for *E. coli* noted that medium components (e.g., Mg^2+^) that increased growth rates also improved subsequent transfer efficiencies^[71]^. It may therefore be possible to customise the recovery broth for a given bacterium by the addition of supplementary components, such as divalent cations, to existing broths and measuring the effect on growth rate. 

The only procedure that appears to universally increase the transfer efficiency of bacterial species is the addition of larger amounts of exogenous DNA to the electroporation buffer. However, obtaining larger amounts of exogenous DNA can be laborious, especially if it requires modification (e.g., appropriate methylation, described below) prior to electroporation. 

### Improving transfer efficiency: conjugation

The factors that affect transfer efficiency tend to be more universal in conjugation than they are in electroporation. Having exogenous DNA that is small in size, facilitating close physical contact between the donor and recipient, and increasing the donor: recipient ratio, generally increase conjugal transfer efficiency in most bacteria. Other factors, such as pH, nutrient starvation and increased incubation time, can increase transfer efficiency but are more species-specific. Artificial modular plasmids are often designed containing no unnecessary DNA to make them as small as possible and improve transfer efficiency. Close physical contact between the donor and recipient can be facilitated by concentrating both bacteria into pellets, resuspending them both in the same small volume of liquid and dotting the solution on the centre of a very dry agar culture plate, upon which both bacteria can grow. Mating of the donor and recipient on a nitrocellulose filter can be used instead of, or in addition to, pellet resuspension. The effects of filter mating on transfer efficiency can also be highly species-specific, with an increase in transfer efficiency observed for one species and complete removal of successful transconjugants for a closely related species^[19]^. 

Another significant advance in the field was the development of well characterised *E. coli* donors^[72,73]^. These strains often possess the genes necessary for the movement of specific mobilisable plasmids. The ability of mobilisable plasmids to replicate in *E. coli *makes them easy to customise, because intermediate plasmid constructs can be electroporated into *E. coli*, multiplied to a high number by plasmid replication and then isolated from a dense cell culture. *E. coli* donors have been used successfully in conjugation with a wide range of phylogenetically diverse bacteria^[46,72,74-77]^ and will likely be very valuable tools in developing genetic manipulation techniques for novel species.

### Circumventing recipient restriction enzymes

The procedures described above are aimed at increasing the amount of exogenous DNA entering the recipient cell. However, once the DNA is inside the recipient cell, it is subject to restriction enzyme activity. Thus, even if exogenous DNA is successfully transferred into the recipient, it may be degraded immediately afterwards. There are several methods allowing exogenous DNA to circumvent or temporarily deactivate this restriction, including heat treatment, methylation, use of intermediate hosts, and plasmid artificial modification (PAM)^[78-80]^. 

**Heat treatment** of recipient cells prior transformation or conjugation can significantly increase transfer efficiency by temporarily inhibiting the activity of restriction enzymes^[76,77,81-83]^. The optimal temperature and duration of heat treatment are highly variable, and should be assessed for a given bacterium by monitoring the number of viable cells remaining after heat treatment at a variety of temperatures and durations. When heat-treated cells are being electroporated, a washing step can be included to reduce the conductivity of electroporation solution, thus preventing arc formation during pulsing^[81]^.

Exogenous DNA can also be protected from restriction by incorporating** site-specific methylation **by commercially available methylases or cell-free extracts of the recipient, prior to transformation. Treatment of exogenous DNA with commercially available methylases increased transfer efficiency in some species^[79,84]^, but considering the complexity and diversity of restriction-modification systems (RMSs) in bacteria, the repertoire of commercially available methylases is unlikely to provide complete protection against all restriction enzymes. An alternative technique involves using the recipient’s own methylases to modify the exogenous DNA by preparing cell-free extracts from the recipient^[78,84]^. Restriction enzymes require divalent cations for activity, whereas DNA methylases do not^[85]^. Thus, EDTA can be added to the cell-free extracts to chelate divalent cations, prior to incubation with exogenous DNA. This treatment endows the exogenous DNA with partial resistance to the recipient restriction activity, resulting in increased transfer efficiency and transformation in previously genetically recalcitrant strains of *Haemophilus *and* Helicobacter*^[78,84]^. When introduced plasmids were reisolated from the transformants, they possessed total resistance to recipient nuclease activity. This raises the possibility that intermediate hosts could be used for *in vivo* DNA methylation of exogenous DNA.

Indeed, the use of *in vivo* modification by related **intermediate hosts** has been the mainstay of genetic manipulation of *S. aureus* for decades^[86]^. The transformation of exogenous DNA into *S. aureus* strains is preceded by passage through the restriction-deficient/modification-proficient *S. aureus* mutant RN4220. This mutant was generated by extensive chemical mutagenesis of the strain *S. aureus* 8325-4^[87]^. However, the isolation of an appropriate mutant by chemical mutagenesis is highly laborious and even then only confers restriction protection against closely related strains of *S. aureus*. Furthermore, many bacterial species do not have the highly conserved RMSs present in *S. aureus*^[86]^. *In vivo* modification by a related intermediate host is also commonly used in lactic acid bacteria^[88,89]^.

The recent boom in the number of publicly available bacterial genome sequences provides an alternative method of *in vivo* modification of exogenous DNA. This method, termed **plasmid artificial modification** **(PAM)**, involves identifying the recipient RMSs *in silico *from the genome sequence, cloning the putative methylation genes into a plasmid and transforming this plasmid into *E. coli*. Exogenous DNA can then be transformed into the recombinant* E. coli* where it is modified *in vivo* by the recipient methylases, and subsequently isolated and electroporated into the recipient. This gives transfer efficiencies comparable to exogenous DNA isolated from the recipient, resulting in 10,000-fold, 1000-fold and 7-fold increases in transfer efficiency for *Bifidobacterium adolescentis*, *Bifidobacterium breve* and *Lactococcus lactis*, respectively^[80,90]^. Expression of recipient methylases from a plasmid with a strong promoter was more effective than expression from the *E. coli* host chromosome^[90]^. As overproduction of methylases can cause cell death of the PAM host^[80]^, the strong promoter should also be tightly regulated, as in the case of pBAD promoters^[91]^. The *E. coli* host itself should be well characterised and lack type IV restriction systems that could target any DNA methylated by the PAM system. It should also lack *dam* and *dcm *genes, as they encode methylases that would create unwanted DNA modifications, which may be targeted by any type IV restriction systems within the recipient. Alternatively, plasmids can also be manipulated to change the DNA sequence at known restriction sites prior to introduction into a new host, improving transformation efficiency^[92]^.

These techniques have been effective at increasing transfer efficiency on their own but could also be used in combination. For example, the effectiveness of a related intermediate host is hindered by the divergence of RMSs between bacteria^[86]^. This diversity means that although related bacteria may possess some conserved RMSs, there are often other RMSs that are strain-specific. Additionally, methylation by cell-free extracts only provides partial protection from restriction activity^[78,84]^. However, if exogenous DNA is passed through a related intermediate strain (giving full protection from conserved restriction enzymes) and then treated with recipient cell-free extracts of the recipient (giving partial protection from strain-specific restriction enzymes), it should increase transfer efficiency for the given strain. This combination approach could prove highly effective in the genetic manipulation of strains whose genomes have not been sequenced. Although PAM appears at first glance to be effective enough to render the other methods obsolete for strains whose genomes have been sequenced, identifying the RMSs in novel species with no closely related reference proteins remains difficult and often requires manual curation of the genome sequence. 

A further complication is that RMSs often exhibit “phase-variable expression” with variation in the coding regions changing the methyltransferase specificity and methylation profiles^[93]^. This feature of bacterial epigenetics allows a bacterium to regulate its gene expression and alter its phenotype, but also affects identification and circumvention of RMSs. Sequencing technologies generating hybrid sequences combining long and short sequence reads provide accurate bacterial genome assemblies^[94]^ that can be screened to identify single nucleotide changes, thus potentially picking up any changes in methylation patterns between bacterial strains.

Improvement of plasmid vectors and counter-selection methods also extends the range of bacterial strains that can be manipulated. The early successful gene transfer experiments with *Bacteroides* spp. were focused on *B. fragilis* and *B. thetaiotaomicron*, with manipulation techniques for other *Bacteroides* spp. remaining much less developed. However, recent advances include developing a range of vectors based on specific polysaccharide utilisation for counter-selection, instead of antibiotic resistance, which enabled the genetic manipulation of multidrug resistant *Bacteroides* strains^[95]^.

### Advanced genetic manipulation techniques

The traditional gene transfer techniques have been augmented in recent decades with the development of a plethora of advanced genetic manipulation methods. Some recent reviews have shown the application of some of these techniques to genetic engineering of probiotic bacteria^[27]^ and marine bacteria^[96]^. This article primarily focuses on the introduction of exogenous DNA into bacteria hosts, rather than the later steps in genetic manipulation. However, we briefly summarise some interesting advances in this area and direct the reader to the cited articles for further information. The majority of these techniques can be divided into two categories: reverse genetics, i.e., selecting genotype (by targeted mutagenesis) and analysing the phenotype; and forward genetics, i.e., selecting a phenotype (from a randomly mutated population) and analysing the genotype.

#### Reverse genetics

Recombineering of bacteria involves the genetic manipulation of recipient DNA by homologous recombination with linear molecules of either ssDNA or dsDNA^[97]^. These DNA molecules possess regions of homology to selected sites in the recipient DNA. On entry into the bacterial cell, the DNA molecules undergo homologous recombination with homologous regions in the recipient DNA. These recombination events are catalysed by a homologous recombination system expressed from a prophage, conjugative transposon or plasmid i.e., λ Red from bacteriophage lambda^[98,99]^, RecE/T from Rac prophage^[100]^ or functional homologues from other bacteriophages^[101]^ or species related to the recipient^[89,101]^. This technique can be used to create insertions, deletions and replacements in recipient DNA. Techniques based on recombineering have been used to rapidly create multiple mutants in the same cell^[102]^ and pools of selected single mutants^[103]^. These techniques are very useful as they enable the analysis of multiple selected genes simultaneously^[97]^. Although powerful methods to study gene function, random transposon libraries require established, efficient DNA delivery and insertion methods, and their use to manipulate Gram-positive gut bacteria is thus far limited to *Bifidobacterium*, *Enterococcus* and bacteria previously classed as *Lactobacillus* species.

Marine bacteria are a potentially rich source of genes with novel functionality, but the same barriers that exist for gut bacteria also exist in developing them through genetic manipulation (reviewed in^[96]^). New tools for the naturally transformable isolates *Vibrio cholerae* and *Vibrio natriegens* include a novel method (multiplexed genome editing by natural transformation, or MuGENT) that generates multiple targeted mutations by simultaneously transforming bacteria with mixed DNA targeting different mutations and coselecting for the removal or introduction of specific phenotypes^[104,105]^. Through this method, either introducing inducible promoters or inactivating expression of nine different genes, mutant *V. natriegens* strains were isolated, producing 100-fold more poly-β-hydroxybutyrate, a storage polymer with potential use as a bioplastic, than parental strains^[106]^.

In addition to the restriction-modification systems mentioned previously, prokaryotes have developed several other defences against incoming foreign DNA such as phage and plasmids^[107]^. One such adaptive immune system involves clustered regularly interspaced short palindromic repeats (CRISPR) and CRISPR-associated genes (CRISPR-Cas). When this protective mechanism is activated, the target DNA is recognised as “foreign”, and interspacer regions based on the sequence of the “foreign DNA” are integrated into the host CRISPR DNA sequence. During RNA synthesis, short CRISPR (cr)RNA molecules are produced that then direct the CRISPR-Cas proteins that initiate degradation of the foreign DNA^[108,109]^. Genome sequence data has enabled identification of multiple CRISPR/cas systems in many diverse bacteria^[27,110]^, although since the type II CRISPR-Cas system has a direct protocol to destroy an incoming DNA sequence, it may be most adaptable. In order to use CRISPR-Cas in gene editing, existing bacterial CRISPR-Cas systems are identified, the spacer motifs and target gene sequences synthesised and integrated into a suitable plasmid, and then transformed into the target host bacterium where the guide RNA targets the required insertion site^[111]^. CRISPR-Cas gene editing has been used successfully to modify the fermentation products of *Clostridium acetobutylicum* from acetone to isopropanol^[112]^ and to abolish tetracycline resistance in *Bifidobacterium animalis* subsp. *lactis*^[113]^. Although CRISPR-Cas has considerable promise as a targeted gene editing tool, specifically altering the genotype and thus phenotype of a target bacterium, it is still essential to have a method to introduce DNA into the target bacterium. 

A modification of the CRISPR-Cas tool has recently been used to perform gene editing in synthetic complex bacterial communities, without culturing individual bacterial strains. This technique combined transposon mutagenesis of an entire microbial community with subsequent targeted DNA insertion using CRISPR-Cas. This ability to edit genomes within complex bacterial ecosystems, without the need to culture members of the community, has huge potential, although further development of the method is required. In a mixed infant microbiota, insertions were only possible in abundant *E. coli* strains, likely owing to the difficulties in introducing foreign DNA into many bacterial genera^[114]^. Additionally, CRISPR-targeted transposons have enabled multiplexed insertions, whereby the same DNA input can be inserted into multiple targeted sites with the recipient chromosome in a single procedure without the need for counterselection^[115]^. 

#### Forward genetics

The development of high-throughput transposon insertional mutagenesis techniques such as transposon directed insertion site sequencing (TraDIS)^[116]^, high-throughput insertion tracking by deep sequencing (HITS)^[117]^, transposon sequencing (Tn-seq)^[118]^, insertion sequencing (INSeq)^[119]^ and genome-wide CRISPR screens (reviewed in^[120]^) has enabled the generation of very large bacterial mutant pools (> 1 million mutants in *Salmonella enterica* serovar Typhi)^[116]^. Massively parallel sequencing technologies^[121]^ have allowed the mapping of enough unique transposon insert sites in these mutant pools to determine the essentiality of every gene in the genome simultaneously^[116-119]^. The genes essential for growth in rich media are first determined. Then this mutant pool can be grown in a variety of *in vitro* (e.g., medium containing bile salts) or *in vivo* (colonisation of mouse) conditions to determine genes that are essential for growth. The level of genome saturation (proportion of the genome containing an insert in the mutant pool) generated by these techniques is sufficient to determine the relative contribution of each gene to growth in the given environment. This is done by comparing the relative abundance of each mutant before and after growth in the experimental growth condition. Mutants that are decreased in relative abundance after experimentation are deemed to be detrimental during growth in the conditions of interest.

## CONCLUSIONS

While recent years have seen significant advances in our understanding of microbial genomics and metabolism, the functions of a large number of genes present in even the most well studied organisms remain unknown. Additionally, large genomic studies providing an abundance of genomic information rely extensively on the homology of queried genes to a relatively small number of experimentally validated genes from a few model organisms. This can lead to the non-annotation or mis-annotation of genes for which there are no close experimentally validated relatives. High-accuracy and high-throughput genetic tools, such as CRISPR and random transposon mutagenesis, offer us the opportunity to determine the functions of much greater numbers of genes than that was possible by traditional genetic manipulation tools such as chemical and ultraviolet mutagenesis. However, even the most advanced tools still require efficient introduction of exogenous DNA to a host bacterium, and optimal conditions for this are still often unknown. Therefore, this first step in genetic manipulation of novel bacteria strains remains a major barrier to detailed investigation of microbial physiology and biochemistry. Nevertheless, bacterial genome sequence information has illustrated the ubiquity of horizontal gene exchange between diverse bacterial species. Thus, the lack of genetic manipulation techniques that target the human gut microbiota may simply be a technical challenge that will be overcome through concerted efforts in the future.

## DECLARATIONS

### Copyright

© The Author(s) 2023.
